# Neoplasia in Cri du Chat Syndrome from Italian and German Databases

**DOI:** 10.1155/2017/5181624

**Published:** 2017-04-24

**Authors:** Andrea Guala, Marianna Spunton, Silvia Kalantari, Ingo Kennerknecht, Cesare Danesino

**Affiliations:** ^1^SOC Pediatria, Ambulatorio di Genetica Clinica, Ospedale Castelli, Verbania, Italy; ^2^Dipartimento di Medicina Molecolare, Università di Pavia and IRCCS S. Matteo, Pavia, Italy; ^3^Institute of Human Genetics, Westfälische Wilhelms-Universität Münster, Münster, Germany

## Abstract

Cri du Chat syndrome (CdC) is a chromosomal abnormality (deletion of short arm of chromosome 5) associated with intellectual disability and typical anatomical abnormalities. Research up to now focuses on the management of the disease during childhood. The longer lifespan of these patients warrants deeper investigations of how and if aging could be affected by the syndrome. We decided to focus on the association of the disease with proliferative disorders. Data on proliferative disorders in a cohort of 321 patients from Italian and German Cri du Chat databases were collected. A neoplasia was present in four patients (age 10–50 yrs), and a fifth patient developed a cholesteatoma during childhood. It is of interest that two cases had an early onset of the neoplasia as compared to the expected age of development in the general population. The chromosome region deleted in 5p does not contain genes whose haploinsufficiency is a well-known main cause of the proliferative disorders observed. We nonetheless believe that reporting even sporadic cases of proliferative disorders in CdC patients may increase our knowledge as to the natural history of the disease. In conclusion, available information suggests that surveillance for cancer development in CdC can follow the guidelines for the general population.

## 1. Introduction

Cri du Chat syndrome (CdC), caused by a deletion involving the short arm of chromosome 5, is observed in 1 : 15.000 to 1 : 50.000 newborns [1]. Nguyen et al. [2] reported a comprehensive review of both clinical and molecular data, suggesting that life expectancy may be normal, in the absence of major malformations. They also report that the oldest person in the 5p Minus Database (USA) including 286 cases is 64 years of age.

For the general population the relation between aging and developing of cancer is well known [3]. As the number of CdC patients surviving into adulthood is increasing, it is of interest to know if the genetic background including the deletion of the short arm of chromosome 5 plays any additional role. These data for CdC patients are completely lacking.

## 2. Results and Case Reports

**Figure 1:**
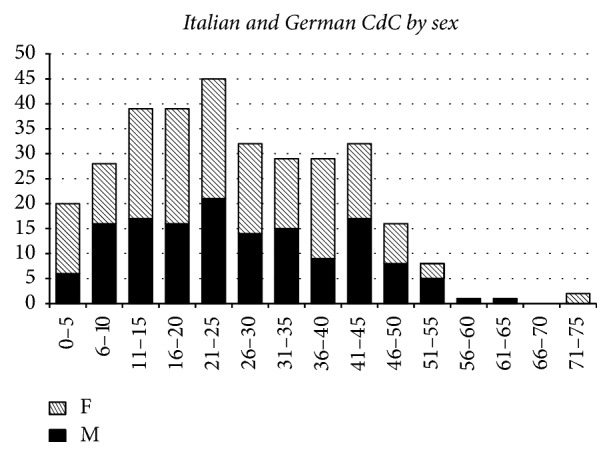
Distribution of 321 Italian and German CdC patients by age (abscissa, five years interval) and sex. F: females and M: males.

A recent analysis of age distribution (January 2017) on the Italian and German Databases for Cri du Chat syndrome is reported in Figure 1.

Among the 321 cases, the observed male to female ratio is 0.83; as far as age is concerned, 60 out of 321 (18.7%) are over 40 years and 3 out of 321 (0.9%) are older than 60 years; the oldest patient is now 74 years old.

Surveillance and management of most clinical problems are largely dependent on self-reporting complaint and discomfort, and CdC cognitive impairment may hamper the possibility of reporting clinical symptoms possibly related to cancer, thus causing an even severe delay in diagnosis and treatment.

We collected data as to the presence of neoplasia in our cohort of cases and we identified four cases in whom neoplasia was diagnosed and a fifth patient who developed a cholesteatoma (Table 1).

### 2.1. Case  1

She underwent surgery at the age of 20 and a 6 cm papillary thyroid cancer was removed; radiotherapy was well tolerated and the patient is disease-free after two years of follow-up. Papillary thyroid cancer is the most common cancer of the thyroid, and the prevalence of the neoplasm is higher in females. It may occur in childhood but is most often seen in adults aged between 30 and 50. Moses et al. [4] demonstrated that multiple genetic alterations may be associated with earlier disease initiation and or progression, and Vriens et al. [5] showed that 6 genes, none of them localized on chromosome 5, had significant differential expression between age groups. Liu et al. [6] demonstrated that promoter mutations in* TERT* (5p15.33) may be relevant to aggressive clinical and pathological characteristics of thyroid cancer.

### 2.2. Case  2

He underwent surgery at the age of 10, but no details as to the type of gastric carcinoid are available; a diagnosis of MEN (Multiple Endocrine Neoplasia, OMIM 131100, 11q13.1) was excluded, and he is alive at follow-up. The average age at diagnosis for gastrointestinal carcinoid tumors is 55 to 65; children rarely develop carcinoid tumors. Molecular genetic studies revealed that the development of neuroendocrine tumors involves different pathways and different abnormalities (point mutations, gene deletions, DNA methylation, and chromosomal losses and/or gains), but no reports suggest any involvement of chromosome 5p [7].

### 2.3. Case  3

She underwent surgery at the age of 50, and the histological diagnosis identifies a ductal carcinoma with necrotic areas, negative for estrogen, progesterone, and HER2/neu. There is no family history of either mammary or ovarian cancer.

### 2.4. Case  4

A breast cancer diagnosis was done when the patient was 31 and she died the same year. The mother of the proband was diagnosed with breast cancer at the age of 52 and died 15 years later. No data concerning the histological type or the eventual presence of mutation on the well-known cancer-associated genes BRCA1/2 are available.

The short arm of chromosome 5 harbors some genes possibly related to the development of breast cancer:* DAB2* (5p13.2) encodes a mitogen-responsive phosphoprotein, downregulated in ovarian carcinoma cell lines, for which a role as tumor suppressor is suggested.* TERT* (5p15.33) encodes a telomerase, a ribonucleoprotein that maintains telomere ends by addition of telomere repeat TTAGGG. Kolquist et al. [8] showed that* TERT* expression is present early during tumorigenesis in vivo and in preinvasive changes in human breast and colon tissues; then it gradually increases during cancer progression.

### 2.5. Case  5

Cholesteatoma is a rare keratinizing stratified squamous epithelium found ectopically in the middle ear, an area characterized by low cuboidal epithelium. It is usually interpreted as a benign condition, but it requires surgical treatment to protect patients from severe complications as temporal bone resorption; some data still support the idea of being a neoplasm, based on the presence of molecular markers of tumor progression [9]. The disease either can be present as congenital or acquired following an otitis media. Exact prevalence is not known but was estimated as 3–12 cases/year/100.000 [10]. The onset of the disease varies according to the etiology: congenital variants are usually diagnosed at 5 years, while acquired forms manifest at 10 years of age, on average. Ecsedi et al. [11] reported that the chromosomal unbalances more frequent in cholesteatoma involve chromosomes 7, 8, and 17; chromosome 5 was not quoted. Tokuriki et al. [12] observed that alterations in gene expression may play a role in the pathogenesis of cholesteatoma, but none of the 12 genes induced or upregulated cholesteatoma was located on chromosome 5p.

## 3. Discussion

No evidence for any increase for cancer has been previously reported in patients affected with CdC [1].

If we take into account the age of development of the proliferative disorders observed, it is of interest that for cases 1 (thyroid papillary carcinoma) and 2 (gastric carcinoid) there is an early onset compared to the expected age of development in the general population. For case 3, we may remark that the incidence of breast cancer at the age of 50 is far lower than at older ages. In case 4, the presence of the neoplasm in two subsequent generations, as well as the very early onset of the disease in the proband, fits the hypothesis of an hereditary form linked to BRCA1/2 genes, and, consequently, an association with CdC is unlikely. In case 5, the patient affected with cholesteatoma, the age of development, was, as expected, in infancy.

CdC is not anymore only a disease of childhood, and survival into adulthood warrants physician's attention to clinical problems seen with aging.

The chromosome 5p region deleted in our cases does not contain genes whose haploinsufficiency is a well-known main cause of the proliferative disorders observed. A recent paper by Espirito Santo et al. [13] demonstrated that, in CdC, if we take into account developmental delay; there is only a weak correlation with the size of the deletion, and many other factors including genetic background, quantitative trait locus polymorphisms, and epigenetic factors must be considered.

We are aware that in our group of patients the cases over 40 years are only 18.7% (60 cases) and thus not representative of the normal population.

However, we believe that reporting even sporadic cases of proliferative disorders in CdC patients may increase our knowledge as to the natural history of the disease and gather useful information for their clinical management.

At present, no evidence is available suggesting recommendations to modify the usual population cancer screening programs in CdC patients.

## Figures and Tables

**Table 1:** List of the five Italian and German CdC patients in whom neoplasia or other benign condition was diagnosed.

Case	Karyotype	Age at diagnosis, yrs	Neoplasia
1	46,XX,del(5)(p14)	20	Papillary thyroid cancer
2	46,XY,del(5)(p14)	10	Gastric carcinoid tumor
3	46,XX,del(5)(p13)	50	Breast cancer
4	46,XX,del(5)(?)	31	Breast cancer
			Other benign condition
5	46,XY,del(5)(p14)	8	Cholesteatoma

## References

[1] Cerruti Mainardi P. (2006). Cri du Chat syndrome. *Orphanet Journal of Rare Diseases*.

[2] Nguyen J. M., Qualmann K. J., Okashah R., Reilly A., Alexeyev M. F., Campbell D. J. (2015). 5p deletions: current knowledge and future directions. *American Journal of Medical Genetics, Part C: Seminars in Medical Genetics*.

[3] Hoeijmakers J. H. (2009). DNA damage, aging, and cancer. *New England Journal of Medicine*.

[4] Moses W., Weng J., Khanafshar E., Duh Q.-Y., Clark O. H., Kebebew E. (2010). Multiple genetic alterations in papillary thyroid cancer are associated with younger age at presentation. *Journal of Surgical Research*.

[5] Vriens M. R., Moses W., Weng J. (2011). Clinical and molecular features of papillary thyroid cancer in adolescents and young adults. *Cancer*.

[6] Liu X., Qu S., Liu R. (2014). TERT promoter mutations and their association with BRAF V600E mutation and aggressive clinicopathological characteristics of thyroid cancer. *Journal of Clinical Endocrinology and Metabolism*.

[7] Leotlela P. D., Jauch A., Holtgreve-Grez H., Thakker R. V. (2003). Genetics of neuroendocrine and carcinoid tumours. *Endocrine-Related Cancer*.

[8] Kolquist K. A., Ellisen L. W., Counter C. M. (1998). Expression of TERT in early premalignant lesions and a subset of cells in normal tissues. *Nature Genetics*.

[9] Lee S. H., Jang Y. H., Tae K. (2005). Telomerase activity and cell proliferation index in cholesteatoma. *Acta Oto-Laryngologica*.

[10] Snow J. B., Wackym P. A. (2009). *Ballenger's Otorhinolaryngology: Head and Neck Surgery*.

[11] Ecsedi S., Rákosy Z., Vízkeleti L. (2008). Chromosomal imbalances are associated with increased proliferation and might contribute to bone destruction in cholesteatoma. *Otolaryngology—Head and Neck Surgery*.

[12] Tokuriki M., Noda I., Saito T. (2003). Gene expression analysis of human middle ear cholesteatoma using complementary DNA arrays. *Laryngoscope*.

[13] Espirito Santo L. D., Moreira L. M. A., Riegel M. (2016). Cri-du-chat syndrome: clinical profile and chromosomal microarray analysis in six patients. *BioMed Research International*.

